# Unusual Presentations of Birth Related Cervical Spinal Cord Injury

**DOI:** 10.3389/fped.2020.00514

**Published:** 2020-09-29

**Authors:** Chien-Chung Lee, I-Jun Chou, Yi-Jung Chang, Ming-Chou Chiang

**Affiliations:** ^1^Division of Neonatology, Department of Pediatrics, Chang Gung Memorial Hospital, Chang Gung University College of Medicine, Taoyuan, Taiwan; ^2^School of Medicine, Graduate Institute of Clinical Medical Sciences, Chang Gung University, Taoyuan, Taiwan; ^3^Division of Pediatric Neurology, Department of Pediatrics, Chang Gung Memorial Hospital, Chang Gung University College of Medicine, Taoyuan, Taiwan; ^4^Division of Pediatric General Medicine, Department of Pediatrics, Chang Gung Memorial Hospital, Chang Gung University College of Medicine, Taoyuan, Taiwan

**Keywords:** spinal cord injury, birth injury, swallowing dysfunction, hoarseness, neonatology

## Abstract

Neonatal spinal cord injury is a rare complication of birth trauma by difficult delivery. The typical manifestations are often catastrophic, include decreased or absent movement, loss of reflexes, apnea or periodic breathing, and a lack of response to painful stimulation. The outcome is usually fatal or severe, with long-term sequelae of respiratory insufficiency, limb weakness, or even paralysis of the limbs. We described a male neonate with a C2 spinal cord injury who was born smoothly by vaginal delivery and was unnoticed initially due to unusual subtle symptoms. He presented with a hoarse voice, swallowing dysfunction, decreased movement of upper limbs, and hypercapnia. After receiving corticosteroid therapy and rehabilitation, he recovered much except that he still needed ventilator support at night.

## Introduction

Neonatal spinal cord injury resulted from birth trauma has been noted since the nineteenth century (1). The exact mechanism of birth related spinal cord injury is unclear, but is believed to be caused by excessive extraction, rotation, or hyperextension of the baby's neck during delivery such as shoulder dystocia, breech vaginal delivery, and difficult deliveries with forceps or vacuum assistance (2–5). The upper and middle cervical spine are injured mostly in vertex delivery, and the lower cervical and upper thoracic region are involved majorly in breech delivery (3). The symptoms and prognosis are dependent on the level of cord and the extent of injury. The clinical manifestations of spinal cord injury are often catastrophic, include decreased or absent movement, loss of reflexes, apnea or periodic breathing, and a lack of response to painful stimulation (3, 6–8). In general, the prognosis of spinal cord injuries is poor with a high mortality rate and long-term morbidities among surviving infants (8, 9). Therefore, early recognition and treatment is crucial to prevent ongoing damage of the injury. However, the clinical suspicion of birth-related spinal cord injury is often delayed, and the diagnosis is challenging due to the fact that symptoms are similar to brachial plexopathy, hypoxic-ischemic injury, and neuromuscular disease (7, 10). Here we described a neonate of high cervical spinal cord injury who presented with unusual subtle symptoms.

## Case Report

A 3,220 g male neonate was born to a healthy primipara woman in the local clinic at gestational age 37 and 3/7 weeks by vaginal delivery without assistance by an instrument. The Apgar scores were 7 at 1 min and 9 at 5 min. The mother had regular antepartum examinations with unremarkable results of prenatal laboratory tests and an ultrasound level II fetal screen. After birth, he was found to have a cephalohematoma on the left parietal scalp, and his upper limbs seemed to move slightly less than his lower limbs. Except for one episode of feeding cyanosis, his general condition was fair. He was discharged from hospital and admitted to a private postpartum care center at 3 days of age. However, he had suffocated milk with cyanosis during the first meal in the postpartum care center and oxygen saturation dropped to 77%. After timely management and oxygen supply, his saturation returned to 95% and he was transferred to our hospital.

After admission, the baby boy had a hoarse voice, but smooth respiration without nasal flaring, chest wall retraction, or abnormal breathing sounds on physical examination. Neurologic examinations showed that his consciousness scale was full, and he had normal muscle power and deep tendon reflex of the lower limbs, but decreased muscle power of the upper limbs (grade 3) and deep tendon reflex (grade 1+). No Erb's palsy, nor Klumpke palsy posture was found. His palmar grasp reflexes and Moro reflex were still present. Laboratory data revealed white blood cells at 8.5 k/μL, hemoglobin at 16.7 g/dL, hematocrit at 47.7%, platelet at 233/μL, and C-reactive protein at 0.58 mg/L. The arterial blood gas showed chronic CO_2_ retentions with metabolic compensate (pH 7.226, PCO_2_ 72.8, PO_2_ 94.1, HCO_3_ 29.5, and base excess 1.9). There were no abnormal findings in both lungs and heart on the chest plain film.

**Figure 1:**
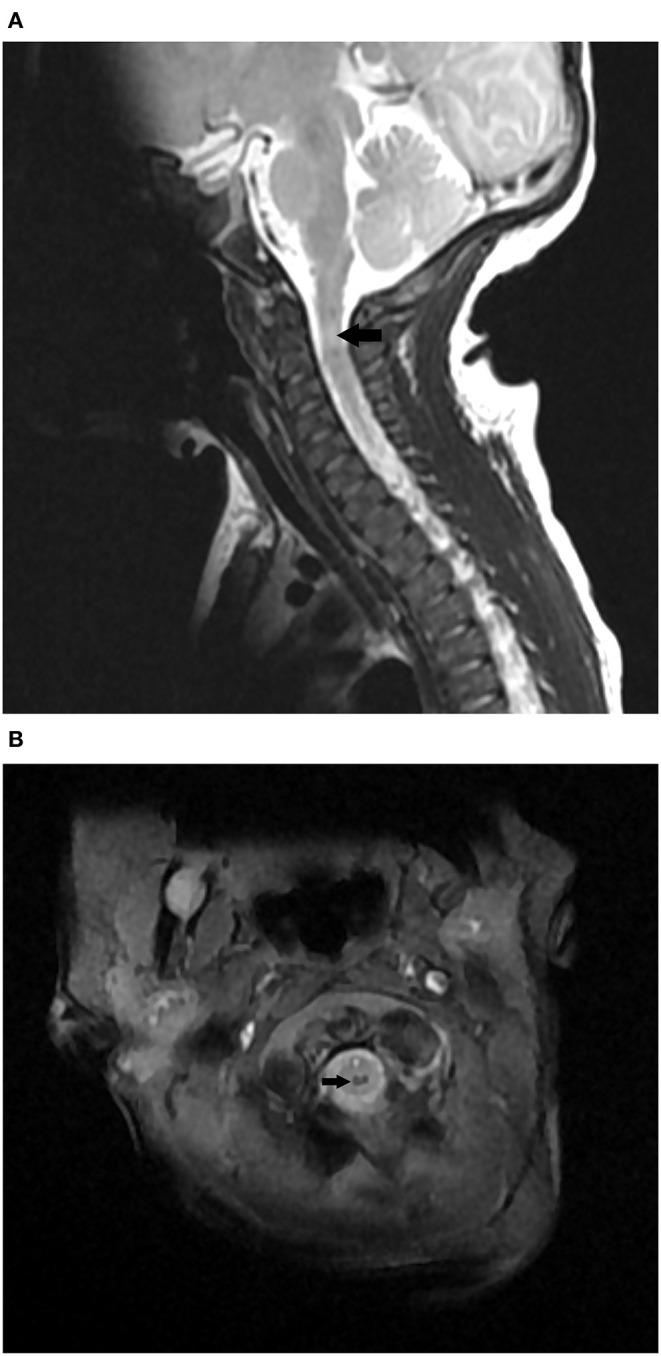
**(A)** MRI Sagittal T2 image showing mixed hypo- and hyper-intensity lesion over the cervical spinal cord at the C2 level. **(B)** MRI axial MERGE image showing hypo-intensity in spinal cord at the C2 level.

A non-invasive intermittent mandatory ventilator was used and an orogastric tube was indwelled. Since he had a hoarse voice, feeding difficulty, and hypercapnia, upper airway anomaly, or vocal cord paralysis was suspected initially. Besides, bilateral brachial plexus injury should also be considered for his upper limbs weakness. Combined with his respiratory depression and neurologic deficits, neuromuscular disorders such as perinatal asphyxia, stroke, anomalies of the central nervous system, congenital myasthenia gravis, and peripheral myopathies were the differential diagnosis. The bronchoscopy was done at 7 days of age to evaluate his upper airway anatomy and vocal cords vibration, and there were no remarkable findings. A head ultrasound was performed at 8 days of age to evaluate problems of the central nervous system, and there were no remarkable findings. He underwent a brain magnetic resonance imaging (MRI) examination on the same day. There was no definite insult of the brain, but a mixed hypo- and hyper-intensity lesion over the cervical spinal cord at the C2 level was found. The MRI scans of the cervical and thoracic spines were done 2 days later, and revealed a hemorrhage 12 × 2 × 1 mm in size at the C2 level without the involvement of other spines (Figure 1). No evidence of brachial plexus injury was found on the brain or spinal MRI scans. The myoglobin level was normal at 19 ng/mL, while the creatine kinase level was slightly elevated to 696 U/L at admission and was decreased to 59 U/L 5 days later. According to his prenatal and postnatal manifestations, birth associated C2 spinal cord injury was highly suspected.

He received dexamethasone 0.15 mg/kg/dose every 6 h for 5 days from 9 days old, every 8 h in the next 2 days, then twice daily in the next 2 days, and once daily in the next 2 days. He also received physical and occupational therapy. After 3 weeks of corticosteroid treatment, he only had cyanotic episodes sometimes when he cried or fell asleep. We tried to remove his ventilator support, but he developed cyanotic episodes frequently. The vein blood gas analysis showed pH 7.247, PCO_2_ 84, PO_2_ 29.6, HCO_3_ 35.8, and base excess 8.5. Therefore, we changed the ventilator strategy and used nasal continuous positive airway pressure support for 8 h at night. With the current therapeutic strategy, he could tolerate well. Follow-up blood gas analysis showed pH 7.358, PCO_2_ 60.4, PO_2_ 59.3, HCO_3_ 33.2, and base excess 7.7. Regarding other symptoms, his voice and movement of upper limbs improved, and he had no swallowing dysfunction as well. He was discharged at 2 months old. One month after discharge, his respiration was stable, and the blood gas showed pH 7.416, PCO_2_ 40.4, PO_2_ 58.2, and HCO_3_ 25.4. Night-time ventilator support was reduced to 4–5 h. At 4 months old, he had good movement of his four limbs, he could reach and grasp objects and rolled to the side. However, mild hypertonia of the limbs with clonus was found on neurologic examinations.

## Discussion

Neonatal spinal cord injury is quite rare, and incidence is estimated from one case per 29,000 birth to one case per 8,000 case (10, 11). Because it is so rare, clinicians may not be so aware of it and ignore the possibility of such birth trauma. Literature about neonatal cervical spinal cord injury are summarized in Table 1 (1, 6, 7, 12–22). Our patient had several characteristics different to previous reports. Our patient was born by uncomplicated vaginal delivery with cephalic presentation. There was no vacuum, nor forceps assistance during delivery. Previous reports have demonstrated that birth-related spinal cord injury usually developed during obstructed labor (Table 1). Only Goetz reported a neonatal spinal cord injury after uncomplicated vaginal delivery (7) and Fazzi reported one after uncomplicated cesarean section following cephalic presentation (23). In addition, unlike previously cases showing catastrophic symptoms of significant dyspnea and paralysis, our case presented with mildly decreased movement of upper limbs, hoarse voice, choking milk at feeding, but relative stable respiration. These subtle symptoms which appeared in the first few days after birth were not able to alert medical staff. Moreover, a hoarse voice and milk choking at feeding were not cardinal symptoms reminiscent of spinal cord injury.

**Table 1:** Reported cases of birth-related cervical spinal cord injury.

**Authors**	**Delivery**	**Lesion**	**Initial symptoms**	**Outcomes (special intervention)**
Shulman et al. (1)	VD with forceps	C1-2	Quadriplegic flaccid No spontaneous breath	Died at 12 h
Pridmore and Aherne (12)	VD with forceps	C2	Quadriplegic flaccid No spontaneous breath	Died at 10 h after ventilator withdraw
Gould and Smith (13)	VD with forceps	C2-3	Quadriplegic flaccid No spontaneous breath	Died at 60 d after ventilator withdraw
Lanska et al. (14), 3 cases	2 VD with forceps, 1 breech VD	1 at medulla-C2 1 at C2-3 1 at C6-7	Quadriplegic flaccid & No spontaneous breath No Moro reflex, No DTR	All ventilator dependent and quadriplegia; One died at 15 mo
Pamphlett and Cala (15)	VD with forceps	C1-2	NA	Died at 7 wk after ventilator withdraw
Menticoglou et al. (6), 15 cases	VD with forceps	All above C4	14/15 Apneic and quadriplegic flaccid	6 death, 7 ventilator dependent, 6 quadriplegia, 1 hemiparesis
Craig and McClure (16)	VD with forceps	Upper cervical	Quadriplegic flaccid No spontaneous breath	Died at 21 d after ventilator withdraw
Moran Newell (17)	Breech CS	C4-5	Quadriplegic flaccid No spontaneous breath No Moro reflex, No DTR	Died at 15 d after ventilator withdraw
Vialle et al. (18)6 of 9 cases	1 VD with forceps 1 shoulder dystocia VD 2 breech VD 1 uneventful VD	2 at C1-2 1 at C2-3 1 at C5-7 1 at C4-5 1 at C6-7	5 quadriplegias 1 monoplegia & Respiratory difficulty	3 died, 3 quadriplegia
Goetz (7)	Uneventful VD	Foramen magnum	Upper limbs flaccid paralysis	Upper limbs flaccid paralysis
UI Haq and Gururaj (19)	Shoulder dystocia VD	C7-T1	Quadriplegic flaccid	Remarkable recovery at 6 mo (corticosteroids treatment)
Montaldo et al. (20)	Shoulder dystocia & VD with forceps	Brain and C1-3	Quadriparesis flaccid No spontaneous breath No DTR	Neurologically normal, except left arm weakness (therapeutic hypothermia)
Yokoi et al. (21)	VD with forceps	lower medulla oblongata- upper cervical cord	Respiratory failure, Hypotonia No primitive reflexes	(Therapeutic hypothermia)
Arnaez et al. (22)	VD with ventouse and forceps failure, Change to CS	Brain and C1-2	Quadriplegic flaccid No spontaneous breath	Diet at 30 d after ventilator withdraw (erythropoietin, therapeutic hypothermia)

*CS, cesarean section; DTR, deep tendon reflexes; VD, vaginal delivery*.

Vocal cord paralysis caused by recurrent laryngeal nerve injury due to birth trauma has been well-recognized as symptoms of stridor, respiratory distress, hoarse cry, dysphagia, and aspiration (8). However, bronchoscopy showed normal vocal cord vibrations in our patient, precluding this possibility. Another possible mechanism of hoarse voice and swallowing dysfunction is velopharyngeal dysfunction caused by pharyngeal plexus injury (24). Pharyngeal plexus lies in the retropharyngeal space between the superior and middle constrictor muscles anteriorly and the longus capitus and colli muscles, prevertebral fascia, and bodies of the second and third cervical vertebrae posteriorly (25). Swallowing dysfunction due to pharyngeal plexus injury has been reported as a risk of cervical spine surgery in adults, and early recognition is crucial to prevent aspiration pneumonia (26). Therefore, we speculated that his hoarse voice and swallowing dysfunction were due to pharyngeal plexus compressed by tissue edema around the second cervical spine. After reviewing the literature, we believe this is the first report of hoarse voice and swallowing dysfunction as symptoms associated with neonatal upper cervical spinal cord injury.

Upper cervical spinal cord injury is often fatal, and the sequelae are serious, but the treatment options are limited. Our patient had been treated with corticosteroids and seemed to recover well. However, it is unclear whether such improvements are because of drugs or the natural course of disease with less severity. UI Hag et al. had reported that one infant received three doses of corticosteroids for perinatal near-total transection of C7 to T1 injury after birth and had remarkable recovery at 6 months of age (19). Since neonatal spinal cord injuries are rare, well-designed pharmacological effect studies are almost impossible. The role of corticosteroids for birth-related spinal cord injury is still uncertain. Recently, therapeutic hypothermia and erythropoietin have been offered to infants with spinal cord injury and the results seemed promising (20–22). However, therapeutic hypothermia needs to be initiated within 6 h of age for beneficial effects. The time window is short, and it is challenging to accurately establish a diagnosis in time. The subtle symptoms shown by our patient make it easy for physicians to miss the time window and fail to initiate a neuroprotective strategy such as therapeutic hypothermia.

In conclusion, birth-related spinal cord injury should be recognized as soon as possible to initiate neuroprotective therapy and avoid further neural damage. Symptoms of neonatal spinal cord injury are often catastrophic but may also be mild with slightly weakened upper limb movement accompanied by a hoarse voice and swallowing dysfunction. All physicians, particularly those who take care of neonates, should be familiar with and alert to symptoms of neonatal spinal cord injury, especially for patients with unusual subtle manifestations.

## Data Availability Statement

The datasets generated for this study are available on request to the corresponding author.

## Ethics Statement

The parents provided written, informed consent to publish this case, including the publication of images.

## Author Contributions

C-CL collected information from the literature and drafted the initial manuscript. I-JC evaluated the patient, provided professional guidance, and revised this manuscript. Y-JC participated in the literature review and assisted in writing part of the initial draft. M-CC provided professional guidance, reviewed, and revised the final manuscript. All authors approved the final manuscript as submitted and agree to be accountable for all aspects of the work. All authors contributed to the article and approved the submitted version.

## Conflict of Interest

The authors declare that the research was conducted in the absence of any commercial or financial relationships that could be construed as a potential conflict of interest.
